# Circular association of hypoglycemia with dementia

**DOI:** 10.18632/aging.101315

**Published:** 2017-10-27

**Authors:** Devi Meyyappan, James S. Goodwin, Hemalkumar B. Mehta

**Affiliations:** University of Texas Medical Branch, Galveston, TX 77555, USA

**Keywords:** hypoglycemia, diabetes, dementia, cognitive decline

Glucose is a primary source of energy for the human brain. Acute or severe hypoglycemia, i.e., depriving the brain of glucose, is associated with neuronal damage in the hippocampus and cerebral cortex [1], two crucial brain components for learning and memory. Therefore, patients with episodes of severe hypoglycemia are often considered at an increased risk for developing dementia, although this issue is controversial. Some studies [2, 3] show an increased risk of dementia in patients with hypoglycemia; however, the Fremantle Diabetes study and Diabetes Control and Complications Trial did not [4, 5]. Mehta et al. address the issue of this association in a study entitled “Association of Hypoglycemia with Subsequent Dementia in Older Patients with type 2 DM” [6].

Mehta et al. conducted a cohort study using the Clinical Practice Research Datalink (CPRD), an electronic medical records data from the United Kingdom, from 2002-2012. The study included patient aged more than 65 years and newly diagnosed with type 2 diabetes mellitus (DM). The analytic cohort included 53,055 patients after excluding patients with no baseline Hba1c number, those who did not receive treatment for DM and those with prevalent dementia. The patients were followed from the date of diagnosis of type 2 DM until they developed dementia, with death treated as a competing risk. Hypoglycemia was modelled as a time dependent variable. The incidence rate of dementia was 19.8 per 1,000 person years among patients with hypoglycemia and 12.5 per 1,000 person years among patients without hypoglycemia. In unadjusted analysis, patients with at least one episode of hypoglycemia had 48% (HR 1.48, 95% CI 1.24-1.76) higher hazards of developing dementia compared with patients with no hypoglycemia episodes. In adjusted analysis which controlled for confounders, hypoglycemia episode was associated with 27% (HR 1.27, 95% CI 1.06-1.51) higher risk of developing dementia compared with no hypoglycemia. The risk also seemed to increase with the number of hypoglycemic episodes.

The strength of this study was the use of the CPRD database, which represented the general UK population. Due to the nature of the data, we controlled for important confounders such as baseline HbA1c, body mass index, alcohol use and smoking status. However, the study may have underestimated of the incidence of hypoglycemia because of underreporting of mild and moderate hypoglycemia. The different stages of dementia were not distinguished. The study did not study the reverse causation, i.e., the risk of hypoglycemia due to dementia. Causal association could not be inferred from this observational study.

The risk ratio obtained in Mehta et al.'s study was lower than that found in prior studies. Whitmer et al. found that hypoglycemia increased the risk of dementia by 44% and Yaffe et al. found a two-fold increased risk [2, 3]. The lower odds ratios could be due to differences in patient populations, the inclusion of newly diagnosed diabetes patients, the duration of follow up, the control of important confounders and statistical methods (use of competing risk models). On the other hand, studies like the prospective Fremantle Diabetes Study in Australians and the Diabetes Control and Complications Trial found no association of hypoglycemia with cognitive impairment [4, 5]. This discrepancy could be because these studies used younger populations in whom dementia was less likely to occur. Our study findings were consistent with a recent meta-analysis that reported a pooled odds ratio of 1.68, suggesting an increased risk of dementia associated with hypoglycemia [7]. In the context of the current evidence from animal studies and observational studies, it is likely that hypoglycemia may be an important risk factor for dementia.

The relationship between dementia and hypoglycemia is bi-directional [3]; that is, hypoglycemia may increase the risk of dementia and dementia patients are more likely to experience hypoglycemic episodes. Cognitive function should be considered in the clinical management of older individuals with DM, especially among patients who have had episodes of hypoglycemia.

## References

[1] Bree AJ (2009). Am J Physiol Endocrinol Metab.

[2] Whitmer RA (2009). JAMA.

[3] Yaffe K (2013). JAMA Intern Med.

[4] Jacobson AM (2007). N Engl J Med.

[5] Bruce DG (2009). Diabetologia.

[6] Mehta HB (2017). J Gerontol A Biol Sci Med Sci.

[7] Mattishent K (2016). Diabetes Obes Metab.

